# Subjective cognitive decline in patients with Parkinson’s disease: an updated review

**DOI:** 10.3389/fnagi.2023.1117068

**Published:** 2023-05-26

**Authors:** Juan Huang, Xingxing Yuan, Lin Chen, Binbin Hu, Lijuan Jiang, Ting Shi, Hui Wang, Wei Huang

**Affiliations:** ^1^Department of Neurology, Second Affiliated Hospital of Nanchang University, Nanchang, China; ^2^Department of Anesthesiology, Changsha Hospital for Maternal and Child Health Care Affiliated to Hunan Normal University, Changsha, China

**Keywords:** subjective cognitive decline (SCD), Parkinson’s disease, cognitive impairment, objective cognitive function, mild cognitive impairment

## Abstract

Cognitive impairment in patients with Parkinson’s disease (PD) worsens the prognosis of PD and increases caregivers’ burden and economic consequences. Recently, subjective cognitive decline (SCD), which refers to self-reported cognitive decline without detectable objective cognitive dysfunction, has been regarded as an at-risk state of mild cognitive impairment (MCI) and a prodromal stage for dementia in Alzheimer’s disease (AD). However, studies on PD-SCD have thus far been scarce, and at present there is no consensus regarding the definition of SCD nor a gold standard as an evaluation tool. The present review aimed to look for an association between PD-SCD and objective cognitive function and found that PD with SCD occurred with brain metabolic changes, which were consistent with early aberrant pathological changes in PD. Moreover, PD patients with SCD were likely to progress to future cognitive impairment. It is necessary to establish a guideline for the definition and evaluation of SCD in PD. A larger sample size and more longitudinal investigations are needed to verify the predictive effectiveness of PD-SCD and to detect earlier subtle cognitive decline before MCI.

## Introduction

Cognitive dysfunction, one of the most common non-motor symptoms (NMSs) in Parkinson’s disease (PD), is up to six times more common in PD than in the healthy aging population, worsens the prognosis of PD, and increases caregivers’ burden and economic consequences (Aarsland et al., 2021). It was estimated that 40–50% of PD presents with mild cognitive impairment (MCI) at baseline, and 75–80% of MCI progresses to dementia in a longitudinal study (Hely et al., 2005; Stuart et al., 2016; Obeso et al., 2017). Recently, the process of cognitive decline in PD patients have received growing interest.

Subjective cognitive decline (SCD) refers to decreases in cognitive capacity without detectable impairment on neuropsychological tests, indicating intact cognitive functions, accompanied by pathological changes, and was believed to be an at-risk state of MCI and a prodromal stage for dementia in Alzheimer’s disease (AD) (Reisberg et al., 2008; Jessen, 2010; Scheef et al., 2012; Jessen et al., 2014; Jack et al., 2018; Wirth et al., 2018). A study showed β-amyloid deposition and atrophy as well as brain activation in people with SCD, suggesting a compensatory mechanism, which might reflect early neuronal dysfunction together with memory performance preserved (Saykin et al., 2006; Rodda et al., 2009; Perrotin et al., 2012). Thus, the state of SCD was recognized as an essential course of AD pathology and a risk factor for cognitive decline (Jessen et al., 2020). Similar to AD, SCD may be an intermediate state between cognitive normality and MCI in PD. Thus, a possible three-stage clinical performance related to cognition might be applicable to patients with PD, with SCD as the prodromal phase, followed by MCI, finally leading to dementia (Erro et al., 2012; Kjeldsen and Damholdt, 2019; Jones et al., 2021; Yoo et al., 2021; Ophey et al., 2022). Neuroimaging study focused on PD patients with SCD (PD-SCD) demonstrated reduced FDG metabolism in the middle frontal, middle temporal, and occipital areas and the angular gyrus of the cortex, which suggested there may be early pathological changes in PD-SCD (Ophey et al., 2022). Follow-up studies showed a significantly higher risk of developing PD-MCI and dementia for patients with PD-SCD compared to PD without SCD at baseline (Erro et al., 2014; Hong et al., 2014b; Galtier et al., 2019; Purri et al., 2020; Jones et al., 2021). However, there are inconsistent results, results just shown a correlation between SCD and depression, anxiety and other related mood features rather than cognitive dysfunction (Lehrner et al., 2014; Santangelo et al., 2014; Baschi et al., 2018; Barbosa et al., 2019). For example, in Barbosa’s study, he regarded SCD as subjective cognitive complaints (SCC), and found SCD severity was related to depression (*p* = 0.026) rather than Montreal Cognitive Assessment (MoCA) scores (*p* = 0.141) in PD with normal cognition (PD-NC), which suggested clinician to alert affective disorder in PD-SCD (Barbosa et al., 2019). Study led by Baschi defined subjective memory complaints (SMC) as SCD, and results showed PD-SCD was significantly associated with anxiety (OR = 3.93) when compared to PD without SCD (Baschi et al., 2018).

Overall, cognitive impairment in PD is a huge financial burden to society as well as caregivers, and it is time to highlight the importance of identifying cognitive decline as early as possible (Aarsland et al., 2021). However, studies on PD-SCD have thus far been scarce, and there is no consensus regarding the definition of SCD nor is there a gold standard as an evaluation tool. Little is known about whether there is a clear association between SCD and cognitive dysfunction or later cognitive decline in PD.

The review aimed to outline how SCD has been used as a diagnostic criterion in studies on PD as well as to describe the possible correlation related to objective cognitive impairment, help recognize SCD as an at-risk state early indicator and allow clinicians to predict conversion to PD-D more accurately.

## Methods

Our aim was to summarize the empirical literature on SCD in patients with PD. We used the following key words: “Parkinson’s disease” (PD) and subjective complaints (“subjective cognitive decline” (SCD), “subjective cognitive impairment” (SCI), “subjective memory complaint(s)” (SMC), “subjective ‘memory impairment” (SMI), or “subjective cognitive complaint(s)” (SCC) to search related literature in the PubMed, Web of Science, and Embase databases. All study designs and articles written in English from 1 January 1970 to 30 April 2023 were included; additionally, related reference lists were carried expand the literature results. Table 1 show the detailed summary of included studies.

**TABLE 1:** Detailed summary of the included studies.

References	Measure for SCD	Sample size	Terms for SCD	SCD domains	Design	Relevant results
Ophey et al., 2022	Subjective Cognitive Decline-Questionnaire (SCD-Q)	30 patients with PD	Subjective cognitive decline (SCD)	Memory, attention, language, executive functions, visuo-cognitive skills, social cognition	Cross-sectional	SCD being an early manifestation of future cognitive decline in PD, and early pathological changes in PD
Yang et al., 2022	Non-Motor Symptoms Scale Domain-5 (NMSs-5) Score ≥ 1	139 *de novo* patients with PD	Subjective cognitive decline (SCD)	Memory, attention	Cross-sectional study	Cognitive domains commonly impaired in PD-SCD were memory and attention; PD-SCD was significantly associated with worse HAMD and HAMA scores.
Rosenblum et al., 2022a	UPDRS-Cognitive Functional features ≥ 1	25 patients with suspected mild cognitive decline; 53 patients without suspected mild cognitive decline; 41 controls	Suspected mild cognitive decline (sMCD)	Memory, language, attention, executive function	Cross-sectional study	PD-sMCD shows higher depression and lower executive function and memory
Rosenblum et al., 2022b	UPDRS-Cognitive Functional features ≥ 1	25 patients with suspected of mild cognitive impairment (sMCI)	Suspected mild cognitive impairment (sMCI)	Memory, language, attention, executive function	Longitudinal study (1 year)	Self-reported cognitive decline may be a marker for identifying gradual cognitive ability decline in PD patients
Galtier et al., 2022	Subjective cognitive decline semi-structured interview	46 patients with PD and 20 controls	Subjective cognitive decline (SCD)	Attention, memory, language, visuospatial functions, executive functions	Longitudinal study (7.5 years)	PD-SCD showed a difficulty for action words
Pan et al., 2021	Cognitive Complaints Interview (CCI)	108 newly diagnosed patients with PD	Subjective cognitive complaints (SCCs)	Memory, language, and visuospatial function	Cross-sectional study	SCCs in early PD with different cognitive status appear to have different pathogenicity; attention/working memory of cognitively normal PD patients with SCCs declined.
Bejr-kasem et al., 2021	Informed by the subject, informant and/or judgment of the site investigator	131 *de novo* PD patients	Subjective cognitive decline	Not indicated	Longitudinal study (5 years)	Patients with minor hallucinations are associated with mid-term subjective cognitive decline.
Xiao et al., 2021	MDS-UPDRS-I 1.1 score > 0	134 patients with late-onset PD (LOPD) 198 patients with early-onset PD (EOPD)	Subjective cognitive complaints (SCCs)	Memory, attention, executive function and orientation	Cross-sectional study	SCCs are only associated with mood disorders in patients with LOPD and SCCs may reflect subthreshold cognitive impairment in the patients with EOPD
Han et al., 2021	MDS-UPDRS-I 1.1 score > 0	189 PD patients with normal cognition (PD-NC) 59 PD patients with SCC (PD-SCC) 135 PD patients with mild cognitive impairment (PD-MCI)	Subjective cognitive complaint (SCC)	Memory, attention, executive function and orientation	Longitudinal study (1–7 years)	PD-SCC patients exhibited faster deterioration of depression than PD-NC patients; PD-SCC showed memory dysfunction compared with PD-NC PD-SCC patients exhibited greater reductions in attention and executive function than the PD-NC group.
Yoo et al., 2021	Cognitive Complaints Interview (CCI)	153 drug-naïve and non-demented PD	Self-awareness of cognitive deficits	Memory, language, and visuospatial function	Cross-sectional	Structural connectivity of frontal lobes is closely associated with SCD in PD. Evaluating frontal structural connectivity from PD-SCD will be important in assessing the actual cognitive.
Siciliano et al., 2021	Parkinson’s Disease Cognitive Functional Rating Scale (PD-CFRS)	90 non-demented patients with PD	Underestimators based on objective–subjective discrepancy	Self-reported impact of cognitive changes on daily functioning	Cross-sectional	underestimation of cognitive performance in PD was associated with the severity of fatigue and depressive symptoms.
Nakhla et al., 2021	Informant Questionnaire of Cognitive Decline in the Elderly (IQCODE)	139 non-demented patients	informant-based cognitive decline	Learning, delayed recall, language, attention, and executive functioning	Cross-sectional	IQCODE was significantly associated with worse objective performance on global cognition, attention, learning, and executive function except for language or visuospatial function
Jones et al., 2021	A participant and/or informant endorsing “cognitive decline.”	483 individuals newly diagnosed with PD	Subjective cognitive complaint (SCC)	Not indicated	Longitudinal study (5 years)	SCC at baseline was not associated with increased risk for future PD-MCI or PDD
Galtier et al., 2021	Subjective cognitive decline semi-structured interview	42 patients with PD and 19 controls	Subjective cognitive decline (SCD)	Attention, memory, language, visuospatial functions, executive functions	Longitudinal study (7.5 years)	PD-SCD patients showed difficulties in vs.-SP functions (executive functions).
Chua et al., 2021	Non-Motor Symptoms Scale Domain-5 (NMSs-5) Score ≥ 1.	121 PD patients	Subjective cognitive complaint (SCC)	Memory, attention	Cross-sectional	PD-SCD is highly prevalent and is associated with emotional factors (depression, anxiety, apathy)
Siciliano et al., 2020	Multifactorial Memory Questionnaire (MMQ)	100 patients with PD	Subjective memory decline (SMD)	Memory	Cross-sectional study	There may be a possible shared pathogenic underlying fatigue and SCD in PD patients
Purri et al., 2020	“Do you feel that your memory and thinking have gotten worse?”	153 PD patients with normal cognition	Subjective cognitive complaint (SCC)	Memory	Longitudinal study (4–5 years)	PD-SCD are more likely to progress to cognitive impairment in long term.
Mills et al., 2020	MDS-UPDRS-I 1.1 score > 0	336 patients with early-stage PD	Subjective cognitive complaint (SCC)	Memory, attention, executive function and orientation	Longitudinal study (>3 years)	PD-SCD were associated with development of PD-MCI over 3 years of follow-up
AlDakheel et al., 2019	MDS-UPDRS-I 1.1 score > 0 Neurobehavioral Inventory (NBI) score >0 or a yes response For General Complaint Question (GCQ)	139 non-demented PD patients	Subjective cognitive complaints (SCCs)	Memory, attention, executive function and orientation	Longitudinal study (1–2 years)	There was no correlation found between PD-SCCs and cognitive impairment; There was no predictive value of PD-SCCs over time
Galtier et al., 2019	Subjective cognitive decline semi-structured interview	43 PD patients and 20 controls	Subjective cognitive decline (SCD)	Attention, memory, language, visuospatial functions, executive functions	Longitudinal study (7.5 years)	PD-SCD is a risk factor for progression to dementia
Barbosa et al., 2019	Non-Motor Symptoms Scale Domain-5 (NMSs-5) Score ≥ 1.	128 PD patients	Subjective cognitive complaint (SCC)	Memory, attention	Cross-sectional	PD-SCD was found to be related to depression, anxiety and apathy
Hong et al., 2018	Cognitive Complaints Interview (CCI)	148 PD with cognitive normality (CN), 71 PD-MCI, and 31 PDD	Subjective cognitive complaint (SCC)	Memory, language, and visuospatial function	Cross-sectional	PD-SCD was related to depression score and was inversely correlated with cognitive performance
Hogue et al., 2018	MDS-UPDRS-I 1.1 score > 0)	351 drug-naive PD patients	Subjective cognitive impairment (SCI)	Memory, attention, executive function and orientation	Longitudinal study (3-year follow-up.)	There was no relationship between PD-SCD and depression, but PD-SCD had lower process speed and visuospatial functions. at baseline
Dupouy et al., 2018	Visual analog scale (VAS)	70 PD patients	Subjective cognitive complaint (SCC)	Memory, executive functions, spatial orientation, attention, and language	Cross-sectional	There was no relationship between SCD and the results of neuropsychological testing.
Baschi et al., 2018	Memory Assessment Clinics Questionnaire (MAC-Q) score ≥ 25	147 PD patients	Subjective memory complaints (SMC)	Memory	Cross-sectional	PD-SCD performs displayed significant lower performance in the MOCA test.
Mills et al., 2016	UPDRSI-1.1 score >0	759 PD patients and 481 controls	Subjective cognitive impairment (SCI)	Memory, attention, executive function and orientation	Cross-sectional	Visuospatial-executive performance and memory had the most significant impact on SCD
Copeland et al., 2016	Existence of any self/informant-reported impairment in 5 cognitive domains	42 patients with PD-MCI	Subjective cognitive impairment (SCI)	Attention, memory, language, visuoperceptual skills, and executive functioning.	Cross-sectional	There was no relation between PD-SCD and cognitive domain
Castro et al., 2016	Existence of any self-reported impairment in memory and/or attention	31 PD without cognitive complaints 21 PD with cognitive complaints 25 controls	Subjective cognitive complaint (SCC)	Attention, memory	Cross-sectional	PD -SCD showed higher scores on HADS PD-without complaints showed poorer cognitive performance.
Koster et al., 2015	A 4-point Likert scale ranging	40 non-demented PD patients and 27 controls	Subjective cognitive complaint (SCC)	Attention, memory, executive functioning and process speed	Cross-sectional	PD-SCD had relationship with attention, executive function, processing speed but not memory
Song et al., 2014	Self-reported memory impairment	30 patients with PD-SCD 47 patients with PD without SCD	Subjective memory impairment (SMI)	Memory	Cross-sectional	PD-SCD group differed PD-without SCD group in MMSE. PD-SCD may be a predictive biomarker of predementia.
Santangelo et al., 2014	Parkinson’s Disease Cognitive Questionnaire (PDCQ),	115 non-demented PD patients	Subjective cognitive complaints (SCC)	Attention, memory, language, visuoperceptual skills, and executive functioning	Cross-sectional	PD-SCD had association with depressive symptoms
Lehrner et al., 2014	Forgetfulness Assessment Inventory (FAI)	104 PD patients 248 controls	Subjective memory complaints (SMC)	Memory	Cross-sectional	Memory tests and depression were significantly correlated to SCD.
Hong et al., 2014b	“Do you feel that you have a declining memory?”	49 PD-SCD 23 controls	Subjective cognitive decline (SCD)	Memory	Cross-sectional	PD-SCD showed poorer performance in visual memory and executive functions and cortical thinning in the frontal, parahippocampal, and posterior cortical areas.
Hong et al., 2014a	“Do you feel that you have a declining memory?”	25 PD-SCD 21 PD without SCD	Subjective cognitive decline (SCD)	Memory	Longitudinal study (2.4-year low-up.)	PD-SCD showed more rapid decline in executive and visuospatial functions and was a risk factor for future cognitive decline
Erro et al., 2014	Item 12 of the non-motor symptoms questionnaire	76 newly diagnosed, untreated patients with PD	Subjective memory complaints (SMC)	Memory	Longitudinal study (2-year low-up.)	SCD were able to predict future development of MCI over 2 years
Uemura et al., 2013	Asking subjects about memory problems	105 PD-naMCI 89 PD-aMCI 99 Dementia 320 Control	Subjective memory complaints (SMC)	Memory	Cross-sectional	SCD was associated with significantly higher scores in depressive symptoms
Hong et al., 2012	“Do you have any memory-related problems?”	20 PD-SCD 15 PD without SCD	Subjective memory complaints (SMC)	Memory	Cross-sectional	PD-SCD had significantly decreased executive functions, process speed as well as decreased gray matter density in the anterior cingulate gyrus and right inferior parietal lobule
Sitek et al., 2011	Self-Rating Scale of Memory Functions (SRSMF)	45 PD patients 33 controls	Self-awareness of memory function	Memory	Cross-sectional	SCD was negatively affected by depressive symptoms.
Benito-León et al., 2011	“Do you suffer from forgetfulness since the last interview?”	46 PD patients 138 controls	Subjective memory complaints (SMC)	Memory	Cross-sectional	PD -SCD had prevalence of 58.7%.
Dujardin et al., 2010	Cognitive complaint interviews (CCI)	25 PD-SCD 25 PD without SCD	Subjective cognitive complaints (SCC)	Memory, language, and visuospatial function	Cross-sectional	PD-MCI and PDD are more frequent among PD-SCD

## Results

### Definition

#### Terminologies for SCD

The current available definition of SCD mainly focused on memory in the context of AD (Jessen et al., 2020). Unfortunately, at present there are no uniform definitions of SCD in PD, and researchers have also used the terms SMD (Siciliano et al., 2020), SMI (Song et al., 2014), SMC (Benito-León et al., 2011; Erro et al., 2012; Hong et al., 2012; Uemura et al., 2013; Lehrner et al., 2014; Baschi et al., 2018), SCI (Copeland et al., 2016; Mills et al., 2016; Hogue et al., 2018), or SCC (Dujardin et al., 2010; Santangelo et al., 2014; Koster et al., 2015; Castro et al., 2016; Mills et al., 2016; Dupouy et al., 2018; Hong et al., 2018; AlDakheel et al., 2019; Barbosa et al., 2019; Purri et al., 2020; Chua et al., 2021; Han et al., 2021; Jones et al., 2021; Pan et al., 2021; Xiao et al., 2021) as descriptions.

#### Assessment tools for SCD

Although patients with PD mainly exhibit severe deficits in executive function, attention and visuospatial function rather than memory (Kehagia et al., 2010), studies have been focused on memory-related questions. “Do you have any memory-related problems?,” “Do you feel that your memory and thinking have gotten worse” or “Have you suffered from forgetfulness since the last interview? “were the questions adopted frequently to define SCD (Benito-León et al., 2011; Erro et al., 2012; Hong et al., 2012; Uemura et al., 2013; Hong et al., 2014a,b; Purri et al., 2020; Lee et al., 2020; Jones et al., 2021). Similarly, some studies applied the UPDRSI 1.1 [(1.1) cognitive impairment] to assess SCD (Mills et al., 2016; Hogue et al., 2018; Mills et al., 2020; Han et al., 2021; Xiao et al., 2021; Rosenblum et al., 2022a,b). Song and Castro defined the decreased self-awareness of attention/memory as SCD (Song et al., 2014; Castro et al., 2016). Siciliano adopted Multifactorial Memory Questionnaire (MMQ) to identify patients with SCD (Siciliano et al., 2020). In Rosenblum’s study, he classified patients as suspected mild cognitive decline (sMCD) based on UPDRS-Cognitive Functional features score ≥ 1 [the mean score of seven MDS-UPDRS items chosen: (1.1) cognitive impairment, (2.1) speech, (2.4) eating, (2.5) dressing, (2.6) hygiene, (2.7) handwriting, and (2.8) doing hobbies], and regarded sMCD as SCD (Rosenblum et al., 2022a,b). Apart from the above studies measuring SCD by simple questions, other studies utilized questionnaires such as the subjective cognitive decline questionnaire (SCD-Q) (Ophey et al., 2022), Cognitive Complaints Interview (CCI) (Dujardin et al., 2010; Hong et al., 2018; Pan et al., 2021; Yoo et al., 2021), Parkinson’s Disease Cognitive Function Rating Scale (PD-CFRS) (Siciliano et al., 2021), Parkinson’s Disease Cognitive Questionnaire (PD-CQ) (Santangelo et al., 2014), Forgetfulness Assessment Inventory (FAI) (Lehrner et al., 2014), Non-Motor Symptoms Scale Domain-5 Score (NMSS-5) (Barbosa et al., 2019; Chua et al., 2021; Yang et al., 2022), Informant Questionnaire on Cognitive Decline in the Elderly (IQCODE) (Nakhla et al., 2021), and Self-Rating Scale of Memory Functions (SRMF) (Sitek et al., 2011). Galtier formulated a subjective cognitive decline semi-structured interview that included seven items (Galtier et al., 2019, 2021, 2022). A visual analog scale was applied by Dupouy et al. (2018) to assess five cognitive domains and helped establish a link between SCD and executive function, language and attention. Koster et al. developed a four-point Likert scale and obtained similar results to those of Koster et al. (2015). Copeland and colleagues took patients as well as their caregivers into account in five cognitive domains, the results showed little agreement between cognitive domains and patients/care partner with subjective reports (Copeland et al., 2016). Bejr-kasem defined patients with SCD according to information informed by the subject, informant and/or judgment of the site investigator, he showed patients with minor hallucinations were associated with mid-term subjective cognitive decline (Bejr-kasem et al., 2021). In order to clarify the relationship between PD-SCD and cognitive performance, AlDakheel adopted 4 methods to measure SCD, MDS-UPDRS-I 1.1 score > 0, Neurobehavioral Inventory (NBI)-subject score >0, NBI-contact score >0, a yes response for General complaint question (GCQ), and he found there were little agreement between SCD and different methods, and no SCD method was associated with cognitive decline (AlDakheel et al., 2019).

Given that patients with PD manifest major impairments in executive function, attention and visuospatial function, not just memory (Kehagia et al., 2010), it is wise for evaluators to assess SCD with questionnaires/problems/interviews including five domains (memory, attention, executive functions, visuospatial functions, language). The definition of SCD should take the functions of these five domains into account, at the same time, the information provided by informant may be contribute to identify PD-SCD. Further research are required to help exploring the application and value of PD-SCD.

### Prevalence

Since there are no guidelines for PD-SCD, researchers have classified PD-SCD differently, and some studies have classified PD-SCD as coexisting with objective impairment. For example, among PD patients in the Dujardin cross-sectional study, 32.22% matched a classification of PD-SCD, of these patients with SCD, 44.83% of PD-SCD met the dementia criteria, of these patients without SCD, just 25.41% met the dementia criteria, suggesting that PD-SCD has a higher probability of manifesting cognitive dysfunction than PD without SCD (Dujardin et al., 2010). Erro reported that approximately 25% of patients with PD underwent SCD, and PD-SCD at baseline may be a risk factor for PD-MCI (Erro et al., 2014). Lehrner studied PD-SCD with FAI and found that 31% of the PD patients reported SCD. Pan showed 30.3% and 12.1% of SCD in PD-MCI, PD-NC, respectively, he found SCD in PD-NC exhibited declined attention/working memory and thought there might be different pathogenicity in SCD with different cognitive status (Pan et al., 2021). Xiao classified patients into early-onset PD (EOPD) and late-onset PD (LOPD), there were 18.66% of EOPD and 24.74% of LOPD reported SCD (Xiao et al., 2021). Siciliano focused on PD with fatigue, and he found the prevalence of SCD was higher in fatigued PD patients when comparing with those with no fatigued (35 vs. 9%) (Siciliano et al., 2020). The PD-SCD evaluated by other studies demonstrated proportions of 32.6% (Baschi et al., 2018), 44.74% (Hogue et al., 2018), 85% (Barbosa et al., 2019), 29.7% (Lee et al., 2020), 23% (Jones et al., 2021), 32.3% (Siciliano et al., 2021), 28.6%, and 30.2% (Galtier et al., 2019, 2021).

Some researchers defined SCD as subjective cognitive impairment without objective cognitive decline. Purri described a 53% incidence of PD-SCD among PD patients with normal cognition and thought PD-SCD may be an indicator of subsequent cognitive impairment (Purri et al., 2020). Hong showed a proportion of 54.3% and found that PD-SCD can predict cognitive decline later (Hong et al., 2014a). Yang found the prevalence of PD-SCD was 28.1% (Yang et al., 2022). Galtier exhibited 30.5% were diagnosed with PD-SCD according to subjective cognitive decline semi-structured interview, and the longitudinal study result showed poor performance in verb naming test in PD-SCD, which suggested the role of linguistic impairment in PD-SCD (Galtier et al., 2022). Other studies demonstrated rates of 16.3% (Lehrner et al., 2014), 22.36% (Hogue et al., 2018), and 27.2% (Baschi et al., 2018).

Above all, there were some variations in the prevalence of PD-SCD among those studies, and the following reasons may be responsible for this. First, different demographic characteristics may contribute to the discrepancy; for example, an older cohort is prone to SCD since cognition declines with age. Second, the tool chosen to assess SCD may also be a factor, and a complete questionnaire may be more accurate than a single item. Third, the definition of SCD used in studies also influenced the results greatly, since PD may have normal memory but impaired planning ability, leading to some people being missed if the focus is only on memory. Finally, a state of anxiety, depression, and apathy in the PD group also accounted for the higher existence of SCD.

### Neuroimaging

Although studies on neuroimaging in PD-SCD have been scarce, the existing results are encouraging. In a study of FDG-PET in PD with normal cognition (*n* = 18 as PD-SCD, *n* = 12 as PD control), results revealed reduced FDG metabolism in the middle frontal, middle temporal, occipital areas, and angular gyrus of the cortex. These regions may be neural correlates of PD-SCD, and could demonstrate an early pathological change in PD-SCD (Ophey et al., 2022), consistent with the finding of hypometabolism in the middle frontal gyrus and inferior parietal lobule in PD-MCI (Huang et al., 2008). Yoo classified PD patients had both CCI score >3 and intact cognition as underestimation of cognitive function, and the group here means to PD-SCD. MRI suggested a close association between the frontal lobes and PD-SCD and thought it important to measure frontal structural connectivity in the early stages of PD (Yoo et al., 2021). Song and colleagues found reduced perfusion in the frontal and inferior temporal cortical regions as well as the anterior cingulate gyrus and thalamus in PD-SCD patients compared to PD patients without SCD, which may provide a potential biomarker of predementia. Other studies by Hong found a decreased gray matter density and cortical thinning in the anterior cingulate gyrus, right inferior parietal lobule and parahippocampal cortices in PD-SCD (Hong et al., 2012, 2014b), suggesting an aberrant PD-related pathology.

The frontal lobe is responsible for executive functions, the temporal area is associated with semantic memory, the anterior cingulate gyrus seems to be relevant to verbal fluency, attention is dominated by the parietal lobe as well as the anterior cingulate gyrus, and the occipital lobe plays a significant role in visuospatial ability (Mohanty et al., 2007; Hong et al., 2012, 2014b; Song et al., 2014; Ophey et al., 2022). The neuroimaging studies above revealed potential neural correlates that underlie PD-SCD, making PD-SCD a promising group to be emphasized in clinician. However, samples from neuroimaging studies to date are relatively small, and larger sample sizes and longitudinal studies are needed for further validation.

### Association with emotion

Because SCD was mainly defined through several subjective questions, the mood features in patients with PD should be taken into account, as we mentioned before. Currently, the relationship between PD-SCD and emotional symptoms was debated, as some studies have described a marked correlation between anxiety, depression or apathy and PD-SCD, while other studies have not shown this. For example, Siciliano et al. (2021) defined underestimators as patients with subjective cognitive complaints but no objective cognitive impairment, they regarded PD-SCD as an underestimator actually and found a positive correlation between underestimator scores and fatigue, depression, and anxiety, suggesting a greater focus on mood features in patients with early PD in the clinical setting. In Yang et al.’s (2022) study, they defined PD-SCD according to NMSs-5 ≥ 1, subsequently, they found PD-SCD was associated with worse Hamilton Depression Scale (HAMD) and Hamilton Anxiety Scale (HAMA) scores. Rosenblum et al. (2022a) regarded PD patients with sMCD as PD-SCD, and he found PD-sMCD showed higher depression when compared to those with no suspected. Chua also obtained the association between PD-SCD and emotional factors (depression, anxiety, apathy) (Chua et al., 2021), and other studies also showed association between PD-SCD and emotional factors (Dujardin et al., 2010; Lehrner et al., 2014; Santangelo et al., 2014; Koster et al., 2015; Castro et al., 2016; Baschi et al., 2018; Barbosa et al., 2019; Purri et al., 2020; Han et al., 2021). At the same time, there still remains unclear between PD-SCD and emotions, since some studies showed no relation between these factors (Hong et al., 2012; Dupouy et al., 2018). According to Baschi, emotional disorders may be both a cause and a consequence of PD-SCD; for example, anxiety in individuals with PD-SCD may be caused by their awareness of cognitive function loss (Baschi et al., 2018). This data reinforced the point that when we encounter PD patients with emotional disorders, it is necessary to incorporate this information to further assess objective cognitive functions. Of course, the discrepancy of the results may be related to the methodology, as age, education, duration and severity of PD influenced moods and should be adjusted.

### Assessment tools for objective cognitive performance

Studies have chosen complete neuropsychological tests for patients with PD, such as the MoCA/Mini Mental State Examination (MMSE) for global cognition, the semantic fluency/trail making test (TMT) for executive function, the word list verbal learning test for memory, the digit span test (DST)/symbol digits modalities test (SDMT) for attention, the naming test for language, and the Rey-Osterrieth complex figure test (ROCFT) for visuospatial ability. Table 2 listed detailed information about neuropsychological tests.

**TABLE 2:** Detailed assessment tools about objective cognitive performance.

References	Global cognition	Attention/working memory	Executive function	Language	Memory	Visuospatial function	Emotional evaluation
Ophey et al., 2022	Mini-Mental State Examination (MMSE); Parkinson Neuropsychometric Dementia Assessment (PANDA); Cognitive Failures Questionnaire (CFQ)	The Digit Span Test (DST)	Wisconsin Card Sorting Test (WCST); Alternating categories sport-fruit	Boston Naming Test (BNT)	Wechsler Memory Scale (WMS)	Block design subset	Beck Depression Inventory (BDI)
Yang et al., 2022	MMSE; Montreal Cognitive Assessment Scale (MoCA).	DST; Trail Making Test A (TMT-A); Stroop Color-Word Test (SCWT)	The Trail Making Test B (TMT-B); Clock Drawing Test (CDT); Animal Fluency Test (AFT)	BNT; the Wechsler Adult Intelligence Scale III (WAIS-III) Similarities Test	Auditory Verbal Learning Test (AVLT); Logical Memory Test (LMT)	Benton’s Judgment of Line Orientation Test (JLOT); the Hooper Visual Organization Test (HVOT)	Hamilton Depression Scale (HAMD) Hamilton Anxiety Scale (HAMA)
Rosenblum et al., 2022a	MoCA Parkinson’s Disease Cognitive Functional Rating Scale (PD-CFRS)		Daily Living Questionnaire (DLQ)	DLQ	DLQ		BDI
Rosenblum et al., 2022b	MoCA PD-CFRS	TMTA	DLQ TMTB	DLQ	DLQ		BDI
Galtier et al., 2022	MMSE			Action generation test (AGT); Anaphora test (APHT); Center-embedded subordinate clauses test (CESCT)			BDI
Pan et al., 2021	MMSE MoCA	DST TMT-A SCWT	TMT-B CDT VFT	BNT WAIS-III	AVLT LMT	JLOT HVOT	HAMD HAMA
Bejr-kasem et al., 2021	MoCA	Symbol Digit Modalities Test (SDMT)	Semantic fluency test	Letter Number Sequencing (LNS)	Hopkins Verbal Learning Test—Revised (HVLT-R)	JLOT	15-item Geriatric Depression Scale (GDS-15); State–Trait Anxiety Inventory (STAI)
Xiao et al., 2021	MoCA						HAMD HAMA
Han et al., 2021	MMSE	SDMT TMT-A	CWT; TMT-B	BNT AFT	AVLT; the Rey-Osterrieth Complex Figure Test (ROCFT)	CFT CDT	BDI
Yoo et al., 2021	MMSE						BDI
Siciliano et al., 2021	MoCA PD-CFRS						BDI; Parkinson Anxiety Scale (PAS); Apathy Evaluation Scale (AES)
Nakhla et al., 2021	An average of the six mentioned composites	Adaptive Digit; Ordering Test–Total; California Verbal Learning Test-II (CVLT-II); D-KEFS CWIT–Color Naming Condition	WCST–Perseverative Responses; D-KEFS CWIT–Inhibition/Switching Condition;	D-KEFS Verbal Fluency–Category; Fluency Total Correct;	CVLT-II; LMT; WMS-III Visual Reproduction II	JLOT; WMS-III Visual Reproduction–Copy	GDS
Jones et al., 2021	MoCA	SDMT	AFT; LNS		HVLT	JOLT	
Galtier et al., 2021	MMSE					JLOT; Facial Recognition Test (FRT); Block design subset	BDI
Chua et al., 2021	MMSE MoCA	WMS-IV Symbol Span; WAIS-IV Digit Span	Fruit fluency tests, frontal assessment battery	BNT; WMS-IV similarities	Alzheimer’s Disease Assessment Scale-Cognitive (ADAS-Cog); ROCF	JLOT Rey-Osterrieth Complex Figure (ROCF) Copy	GDS Apathy Scale (AS); Hospital Anxiety and Depression Scale for Anxiety (HADS-A)
Siciliano et al., 2020	PD-Cognitive rating scale						BDI PAS AES
Purri et al., 2020	MoCA Mattis Dementia Rating Scale-2 (MDRS-2)	TMT-A SDMT	LNS phonemic verbal fluency; semantic verbal fluency (animals) TMT-B	BNT	HVLT-R	JLOT; CDT	GDS-15
Mills et al., 2020	MoCA						BDI
AlDakheel et al., 2019	MMSE	Delis Kaplan Executive Function System (DKEFS) Color Word; Interference Color Naming test WMS-III letter-number sequencing test,	Visual Verbal Test TMT-B	DKEFS Verbal Fluency; Category Fluency test; BNT	RCFT; CVLT-II	JLOT; Copy Trial of the RCFT	
Galtier et al., 2019	MMSE	DST	WCST (categories) letter fluency		CVLT; Spatial Recall Test (SRT)	JLOT; Block design	BDI
Barbosa et al., 2019	MoCA						Hospital Anxiety and Depression Scale (HADS); Apathy Scale.
Hong et al., 2018	Korean version of the Mini Mental State Examination (K-MMSE) Korean version of the Montreal Cognitive Assessment (K-MoCA)	TMT-A DST	Semantic fluency (animal); CDT	K-BNT; Word similarity	Delayed recall in Seoul Verbal Learning Test (SVLT)/RCFT	Copying task of RCFT; Clock copying (CLOX2)	BDI
Hogue et al., 2018		SDMT WAIS- III Letter Number Sequencing subtest	Letter number sequencing	Semantic fluency (animals)	HVLT	JLOT	GDS-15
Dupouy et al., 2018	MATTIS dementia rating scale.	TMT (B-A) SCWT, SDMT; Digit span; Benton visual retention test	WAIS-III-SDMT; semantic/phonemic verbal fluencies	Semantic/phonemic verbal fluencies; the ExaDé confrontation naming test	free and cued selective reminding test (FCSRT); RCFT	RCFT; Visual Object and Space Perception Battery (VOSP)	Hamilton Anxiety and Depression Scale(HADS)
Baschi et al., 2018	MMSE MoCA	Visual search TMT-A	Frontal Assessment Battery Raven Colored Progressive Matrices	Aachener Aphasie Test naming; Token Test;	Rey Auditory Verbal Learning Test (R-AVLT); Story Recall Test	Constructional Apraxia; CDT	HADS
Mills et al., 2016	MoCA						
Copeland et al., 2016		WMS–III DST; TMT-A	WCST; SCWT Interference Task score	BNT; semantic fluency (Animals)	Visuospatial Memory Test; HVLTR; LMT	JLOT; HVOT	
Castro et al., 2016	Parkinson’s disease–cognition (SCOPA-COG)		TMT-B	Phonemic verbal fluency; BNT		CDT	Hospital anxiety and depression scale (HADS) BDI
Koster et al., 2015		SCWT; DST; SDMT	Letter Fluency; Letter-Number Sequencing	Animal fluency		CVLT-II	Minnesota Multiphasic Personality Inventory-2 Depression/Psychasthenia
Song et al., 2014	MMSE						
Santangelo et al., 2014	MoCA						BDI
Lehrner et al., 2014	MMSE	TMT-(B-A)	TMT-A SCWT	Modified Boston Naming Test (mBNT); Semantic verbal fluency	Verbal Selective Reminding Test (VSRT)		BDI
Hong et al., 2014b	K-MMSE	DST; SCWT	Phonemic fluency Semantic fluency	k-BNT	SVLT; Visual memory	RCFT copy	BDI
Hong et al., 2014a	K-MMSE	DST; SCWT	Phonemic fluency Semantic fluency	k-BNT	SVLT; Visual memory	RCFT copy	BDI
Erro et al., 2014		Frontal assessment battery; TMT (B-A)	TMTB; SCWT; Phonological/semantic fluency task		R-AVLT	JLOT; CDT	HADS
Uemura et al., 2013	MMSE						GDS-15
Hong et al., 2012	k-MMSE	DST	Go-no-go test Contrasting program	K-BNT	SVLT	RCFT	BDI
Sitek et al., 2011	MMSE	SCWT			AVLT		BDI
Benito-León et al., 2011	37-item version of MMSE		TMT	Animals/fruits Naming test	Story recall test		
Dujardin et al., 2010	MMSE	DST; SCWTt	Letter/number sequencing	Word-generation task	Buschke 16-item recall test Free recall		Montgomery and Asberg depression rating scale (MADRS)

### Association with objective cognitive functions in cross-sectional study

Lehrner was interested in the relationship between cognitive complaints and cognitive impairment, he found significant correlations between PD-SCD and worse MMSE performance (Lehrner et al., 2014). Hong et al. (2014b) focused PD-SCD on memory impairment, he investigated the cognitive performance and cortical thickness (*n* = 49 PD-SCD, *n* = 23 controls), results showed PD-SCD have poorer performance in visual memory and executive functions, which was consistent with cortical thinning in frontal and posterior cortical areas, since frontal and posterior regions responsible for executive function and visual function, respectively. In Song et al.’s (2014) study, PD-SCD group (*n* = 30) differed PD-without SCD (*n* = 47) in global cognition measured by MMSE and suggested PD-SCD may be a predictive biomarker on predementia. Nakhla et al. (2021) took informant-based responses into account and measured them by IQCODE and ultimately found that higher scale scores were negatively correlated with objective performance, including attention, executive function, memory and global cognition. Hong et al. (2018) assessed PD-SCD by CCI and suggested that an increasing score was strongly correlated with poorer objective cognitive functions (global cognition and all five cognitive domains) after adjusting for a depressive score. Baschi found a lower MoCA score in PD-SCD patients than in PD patients without SCD (Baschi et al., 2018). Regression models analyzed by Mills et al. (2016) suggested lower scores on memory, executive and visuospatial functions in PD-SCD. Koster et al. (2015) showed that patients with PD have higher proportion of self-reported difficulties in attention and executive functions but not memory. Dujardin reported more objective cognitive dysfunction among PD patients with cognitive complaints than among PD patients without SCD (Dujardin et al., 2010). In a word, studies referred above showed a relationship between SCD and decreased cognitive manifestation, while the remaining reported mixed results (Castro et al., 2016; Copeland et al., 2016; Dupouy et al., 2018). Castro obtained a contradictory conclusion with the above results. In this study, PD with cognitive complaints performed at a higher level, suggesting better cognitive status (Castro et al., 2016). Additionally, Dupouy and Copeland found no association between neither the patient’s nor the caregiver’s complaints and the patient’s objective cognitive manifestation (Copeland et al., 2016; Dupouy et al., 2018).

Attention and executive functions, which have neural correlates with the parietal and frontal lobes, were the most reported to have a strong correlation with the existence of PD-SCD, and these findings were consistent with the pathological changes in the early stage of PD. Some studies also found that PD-SCD was related to memory, and we think the results were affected by the assessed tools used as well as the targeted population. On the one hand, studies defined SCD as self-reported memory complaints, and the association investigated may be memory rather than other domains. On the other hand, studies comparing PD-SCD and PD-MCI concurrently may increase the correlation with one another. Dupouy and Copeland found no association between SCD and objective cognitive performance, which may be due to the assessment used or the psychiatric symptoms among their cohort. Overall, the association between SCD and objective cognitive performance remains unclear now. SCD are subjective and may be influenced by countless factors and the conflicting results obtained can be associated with measurements of cognitive status, definition of SCD, sample size, study design and so on. Thus, it is difficult to show a clear relationship between the two, especially when the methods applied are not precise or extensive enough to evaluate SCD or objective cognitive functions.

### Association with objective cognitive functions in longitudinal study

In Jones et al.’s (2021) study (*n* = 483 PD patients), a single item was used to assess PD-SCD, and the results revealed no relation between PD-SCD at baseline and PD-MCI/PDD 5 years later. A longitudinal study (*n* = 139 non-demented PD patients) adopted 4 methods to elicit PD-SCD, it explored the link between PD-SCD and cognitive performance subsequently, results here showed there was no correlation between PD-SCD and cognitive decline and no predictive value of PD-SCD over time (1–2 years) (AlDakheel et al., 2019). Conversely, a follow-up study by Galtier suggested that PD-SCD patients (36.4%) are at higher risk of converting to dementia than PD without SCD (14.3%) 7.5 years later, and the PD-SCD group showed impairments in visuospatial and visuoperceptual functions, which was linked to thinning of posterior cortices (Galtier et al., 2021), at the same time, PD-SCD showed poor performance in verb naming test in the same longitudinal study (*n* = 46 PD, *n* = 20 controls), which suggested the possible linguistic dysfunction in PD-SCD (Galtier et al., 2022). Rosenblum followed up PD-sMCD (*n* = 25) and found self-reported cognitive decline may be a marker for predicting cognitive ability decline after 1 year (Rosenblum et al., 2022b). Han and colleagues regarded PD-SCD as an intermediate status of PD-NC and PD-MCI, they divided people into three groups: PD-NC (*n* = 189), PD-SCD (*n* = 59), PD-MCI (*n* = 135), the longitudinal study (1–7.5 years) found PD-SCD showed memory impairment and greater reductions in attention and executive function compared with PD-NC, which suggested PD-SCD may be high risk individuals progression to cognitive decline later (Han et al., 2021), and the results were similar to Hong’s and Purri’s study (Hong et al., 2014a; Purri et al., 2020). In Purri’s study (*n* = 153 PD patients with normal cognition), they defined PS-SCD as subjective cognitive complaints with objective cognitive normality and found that the PD-SCD group performed worse on global cognition, executive function and processing speed than the PD without SCD group. Additionally, PD-SCD more easily converted cognitive impairment longitudinally, indicating that PD-SCD may be a warning of subtle cognitive decline (Purri et al., 2020). A larger longitudinal study (*n* = 336 patients with PD) led by Mills et al. (2020) showed that PD-SCD patients were more likely to develop PD-MCI over 3 years of follow-up and can be used to predict future cognitive impairment, which was similar to other studies (Hong et al., 2014a; Galtier et al., 2019). In Hong et al.’s (2014a) study, the findings were consistent with the conclusion that PD-SCD may be a risk factor for future cognitive dysfunction, since their results showed that PD-SCD could predict progression to decreased cognition 1–4 years later. Erro et al. (2014) also focused on the power of PD-SCD to predict further cognitive decline, he found patients with subjective memory decline at baseline (*n* = 76) were independent predictor, which can predict the progression to MCI with 2 years longitudinal study, Hogue et al.’s (2018) conclusion was similar to Erro, he created a regression model included SCD in baseline, and the model can predict future cognitive decline, which showed encouraging predictive value of PD-SCD.

In summary, the majority of the above studies considered PD-SCD to be risk factor for impaired objective cognition, and PD-SCD may be a potential way to identify early cognitive decline. However, some problems should be noted. Most studies evaluated PD-SCD with a single item partly concerned with memory, which could eventually influence the results, thus, assessing PD-SCD according to more comprehensive cognitive domains may contribute to the consistency of subjects selected. Secondly, emotional factors should be emphasized and adjusted when exploring the correlation between cognitive performance and PD-SCD, because emotional factors influenced cognitive function and may be linked with PD-SCD, which interfered the results. Thirdly, it may be potential way to associate longitudinal study with more neuroimaging researches in PD-SCD.

## Conclusion

Subjective cognitive decline has been gaining increasing interest in recent years. Most of the results reported that patients with PD-SCD had a correlation with objective cognitive decline, and longitudinal studies also revealed a predictive risk for future cognitive dysfunction. Neuroimaging supported the above studies in terms of neural correlates as well as brain metabolism.

However, there are still some problems that need to be solved as soon as possible. First, it is necessary to establish a consensus on PD with SCD. According to the SCD consensus on patients with AD, we recommend that the definition of PD-SCD should be purely self/informant-reported cognitive decline, and this definition may contribute to proving a pre-MCI stage of subtle cognitive decline. Next, studies used different methods to evaluate SCD, and assessments of SCD ranged from a single item to several face-to-face questions and a complete questionnaire. Given the specific characteristics of cognitive impairment in PD, a questionnaire including all five domains should be adopted. Additionally, information derived from caregivers may be useful to detect PD-SCD. Finally, the sample size should be larger, and more longitudinal investigations are needed to verify the predictive effectiveness of PD-SCD.

Overall, a clear definition of PD-SCD would help identify earlier stages of cognitive impairment before PD-MCI, explore more risk factors associated with the existence of PD-SCD and allow for early attention and intervention in subtle cognitive decline stages, which may ultimately reduce the burden of cognitive impairment on society and caregivers.

## Author contributions

JH had the idea for the manuscript. WH critically revised the work. All authors contributed to the study conception and design, performed the literature search, data analysis, and drafted the manuscript.
